# Molecular Pathways and Key Genes Associated With Breast Width and Protein Content in White Striping and Wooden Breast Chicken Pectoral Muscle

**DOI:** 10.3389/fphys.2022.936768

**Published:** 2022-07-08

**Authors:** Martina Bordini, Francesca Soglia, Roberta Davoli, Martina Zappaterra, Massimiliano Petracci, Adele Meluzzi

**Affiliations:** ^1^ Department of Agricultural and Food Sciences (DISTAL), Alma Mater Studiorum—University of Bologna, Bologna, Italy; ^2^ Department of Agricultural and Food Sciences (DISTAL), Alma Mater Studiorum—University of Bologna, Cesena, Italy

**Keywords:** white striping, wooden breast, WGCNA, functional analysis, gene network

## Abstract

Growth-related abnormalities affecting modern chickens, known as White Striping (WS) and Wooden Breast (WB), have been deeply investigated in the last decade. Nevertheless, their precise etiology remains unclear. The present study aimed at providing new insights into the molecular mechanisms involved in their onset by identifying clusters of co-expressed genes (i.e., modules) and key loci associated with phenotypes highly related to the occurrence of these muscular disorders. The data obtained by a Weighted Gene Co-expression Network Analysis (WGCNA) were investigated to identify hub genes associated with the parameters breast width (W) and total crude protein content (PC) of *Pectoralis major* muscles (PM) previously harvested from 12 fast-growing broilers (6 normal vs*.* 6 affected by WS/WB). W and PC can be considered markers of the high breast yield of modern broilers and the impaired composition of abnormal fillets, respectively. Among the identified modules, the turquoise (*r* = -0.90, *p* < 0.0001) and yellow2 (*r* = 0.91, *p* < 0.0001) were those most significantly related to PC and W, and therefore respectively named “protein content” and “width” modules. Functional analysis of the width module evidenced genes involved in the ubiquitin-mediated proteolysis and inflammatory response. GTPase activator activity, PI3K-Akt signaling pathway, collagen catabolic process, and blood vessel development have been detected among the most significant functional categories of the protein content module. The most interconnected hub genes detected for the width module encode for proteins implicated in the adaptive responses to oxidative stress (i.e., *THRAP3* and *PRPF40A*), and a member of the inhibitor of apoptosis family (i.e., *BIRC2*) involved in contrasting apoptotic events related to the endoplasmic reticulum (ER)-stress. The protein content module showed hub genes coding for different types of collagens (such as *COL6A3* and *COL5A2*), along with *MMP2* and *SPARC*, which are implicated in Collagen type IV catabolism and biosynthesis. Taken together, the present findings suggested that an ER stress condition may underly the inflammatory responses and apoptotic events taking place within affected PM muscles. Moreover, these results support the hypothesis of a role of the Collagen type IV in the cascade of events leading to the occurrence of WS/WB and identify novel actors probably involved in their onset.

## Introduction

The intensive breeding programs implemented in the last half-century to improve broilers’ production traits have been accompanied by the appearance of severe conditions mainly affecting the *Pectoralis major* muscle (PM) of fast-growing and high breast-yield hybrids (Lake and Abasht, 2020). These conditions include the White Striping (WS) and Wooden Breast (WB) defects (Petracci et al., 2019) which are known as “growth-related abnormalities,” as their occurrence is strongly connected to the high breast yield reached thanks to the selection programs carried out to develop the modern chicken hybrids (Kuttappan et al., 2013; Clark and Velleman, 2016). These conditions are responsible for detrimental alterations at the microscopic level. Indeed, the affected pectoral muscles exhibit extensive muscle degeneration which results in lower crude protein contents in the forthcoming meat (Wold et al., 2017; Dalle Zotte et al., 2020; Xing et al., 2020; Oliveira et al., 2021). Due to their adverse impact on meat quality and consumer acceptability (Kuttappan et al., 2012; Oliveira et al., 2021), these defects are responsible for significant economic losses for the poultry meat industry (Baldi et al., 2018; Zanetti et al., 2018). Indeed, severe degrees of these myopathies determine negative effects on the technological and nutritional properties of chicken meat, as suggested by the occurrence of lipid infiltration, collagen deposition, and protein degradation characterizing abnormal fillets (Tasoniero et al., 2016; Baldi et al., 2018; Huang and Ahn, 2018). Although characterized by distinctive phenotypes (Kuttappan et al., 2016), these muscular abnormalities share similar microscopic features (Petracci et al., 2013; Sihvo et al., 2014), thus suggesting the existence of potentially common causative mechanisms (Soglia et al., 2021). Briefly, fillets affected by WS are characterized by the appearance of white striations parallel to the fiber direction, while the WB-affected ones show the presence of hard, pale, and out-bulging areas mainly in the cranial and/or caudal area of breast muscle (Baldi et al., 2018; Huang and Ahn, 2018).

In this context, notable efforts have been made to discover the causal factors of these growth-related abnormalities. Several years of studies led to an extensive description of phenotypic and molecular perturbations that characterize these abnormalities and that may underly their occurrence. Some speculations have been made to try elucidating the mechanisms underlying these defects (Boerboom et al., 2018; Lake et al., 2020; Velleman, 2020; Ayansola et al., 2021). Among them, recent studies have highlighted a possible role of the development of oxidative stress (Hubert and Athrey, 2022), mitochondrial dysfunction (Papah et al., 2017), and energy metabolism dysregulations (Papah and Abasht, 2019) in the cascade of events triggering the occurrence of WS and WB. Several studies focused also on the decreased vascularization of modern broilers’ PM (Kuttappan et al., 2013; Lake and Abasht, 2020), which may result in hypoxic conditions due to the impaired oxygen supply at the muscular level, possibly triggering or exacerbating the WS and WB myopathic conditions (Soglia et al., 2019). More recently, it has been suggested that the endoplasmic reticulum stress caused by an accumulation of misfolded proteins may be one of the first phenomena leading to the development of these myopathic conditions (Bordini et al., 2021; Soglia et al., 2021). However, despite the considerable increase in the knowledge regarding the complex etiology associated with the development of these defects, the precise factors triggering the cascade of events leading to their occurrence remain to be elucidated. In this regard, there is still a need to further explore the pathophysiological mechanisms underlying the progression of these myopathic conditions.

Over the past decades, great strides in new high-throughput technologies have provided the scientific community with a remarkable opportunity to investigate the genetic architecture and the regulatory mechanisms of complex traits and diseases (Rotival and Petretto, 2014). In particular, co-expression network analysis has rapidly become a prevalent and powerful approach to elucidate the specific molecular processes underlying physiological mechanisms and pathological pathways (Hocquette et al., 2009; van Dam et al., 2018). Currently, Weighted Gene Co-expression Network Analyses (WGCNA) have been successfully employed in the study of various human diseases, most notably in different cancer research (Bai et al., 2020; Jia et al., 2020; Chen et al., 2021), to identify patterns of co-expressed genes (i.e., modules) associated with specific disease features. Combining the identification of key gene modules with hub gene analyses has proved to be useful in detecting molecular mechanisms and candidate genes that may be at the base of the physiological changes characterizing different human and animal disorders (van Dam et al., 2018). In our previous work performed on 52-day-old broilers (Bordini et al., 2021), WGCNA analysis evidenced pathways involved in the extracellular matrix organization, collagen metabolism, and unfolded protein response as some of the most significant functional categories probably involved in the cascade of biological reactions that result in onset of the growth-related myopathies. Moreover, the Collagen type IV (COL4) coding genes were found as the most significant hubs. Considering the strong similarities between the histological and molecular features that characterize COL4-related disorders in humans (Guerci et al., 2012) and the growth-related myopathies in broilers, COL4 genes may be interesting candidates possibly involved in the onset and/or progression of events leading to WS and WB abnormal conditions. However, the approach used in our previous work did not allow us to explore gene networks and hub genes possibly associated with phenotypes not yet investigated and significantly related to the broiler breast abnormalities occurrence. Therefore, for the present study, we decided to consider and investigate two traits that strongly characterize the WS/WB emergency in fast-growing chickens: the first one directly related to the breast dimensions of modern hybrids, the breast width, and the second one related to one of the major meat quality traits of affected breasts, the crude protein content.

Within this context, this study aimed at providing new insights into the molecular basis underlying the onset of WS/WB through the analysis of key modules and hub genes associated with breast width and total crude protein content of broiler PM: two traits chosen as indicative markers of the broiler breast yield and the impaired composition of the affected breasts, respectively. In particular, our study aims to further investigate the gene expression networks involved in the biochemical and physiological changes characterizing broiler breast muscles affected by WS and WB. As far as we know, this is the first study trying to investigate co-expression patterns and hub genes associated to the protein content and breast width of fast-growing chickens’ pectoral muscles.

## Materials and Methods

### Data Collection and Co-Expression Network Analysis

The present study was carried out by using the set of samples collected in our previous research (Zambonelli et al., 2016). This set of samples consisted of 12 *Pectoralis major* muscles (6 macroscopically normal vs*.* 6 severe WS/WB) belonging to 52-day-old fast-growing broilers selected from the same flock of Ross 708 (males, weighing around 3.7 kg), and slaughtered in a commercial abattoir. A detailed description of samples’ selection and preparation is reported in Zambonelli et al. (2016). Briefly, 49 meat quality parameters (including technological and morphological traits as well as those related to the proximate composition of samples) were analyzed together with the microarray profiles (18,308 probes) of the 12 broiler breast fillets. The same phenotypic and gene expression data have been used in the present research to perform the Weighted Gene Co-expression Network Analysis, using the “WGCNA” package (Langfelder and Horvath, 2008) in R environment (R Core Team., 2020).

The gene expression profile of each sample was used to construct the co-expression network by creating an adjacency matrix in which the nodes correspond to the gene expression level and the edges between genes are represented by their pairwise correlation (calculated using the Pearson’s correlation coefficient), as previously described by Zappaterra et al. (2021). In particular, to establish the adjacency matrix, an appropriate soft threshold power (β value) of 10 was chosen applying the approximate scale-free topology criterion (Zhang and Horvath 2005; Langfelder and Horvath 2008). The topological overlap matrix (TOM) and the corresponding dissimilarity (dissTOM = 1-TOM) were used to construct the adjacency matrix (Langfelder and Horvath, 2008). Finally, the blockwiseModules R function was used to build the network of gene co-expression patterns with the dissTOM values used as the distance measure for the gene hierarchical clustering. In particular, the latter allowed us to identify groups of co-expressed genes (i.e., modules) by employing the Dynamic Tree Cut algorithm (Langfelder and Horvath, 2008).

### Module-Trait Association Study

Once the list of gene modules (i.e., groups of highly interconnected genes) had been identified and named using different color labels, the module eigengene (ME) of each module was calculated using the principal component analysis criterion to assess the relationship between modules and traits (Langfelder and Horvath, 2008). In fact, the ME represented a weighted average expression level of the considered module, and it was used to identify the “module-trait association” by calculating the Pearson’s correlation between every ME and trait.

### Identification of the Most Significant Traits and Modules

In the current research, two traits measured in Zambonelli et al. (2016) and not yet considered in our previous study (Bordini et al., 2021), were investigated to deepen the knowledge on the molecular mechanisms underlying WS/WB abnormalities in broiler breast meat. For this purpose, in the present study, width (expressed in mm; W) and protein content (expressed in %; PC) of PM were considered in light of their highly significant association with the onset of WS and WB myopathies (*p* < 0.0001) (Zambonelli et al., 2016) and their relevance as phenotypic markers of the high breast yield and impaired muscle composition, respectively.

For the next steps of our analysis, we focused on those modules identified by WGCNA that showed the highest absolute value of module-trait correlation with the considered traits (i.e., W and PC). For each selected module, the software calculated the gene significance (GS; i.e., the gene-trait correlation value) and module membership (MM; i.e., the intramodular connectivity) of genes belonging to the selected modules (Langfelder and Horvath, 2008).

### Functional Enrichment Analyses of Selected Modules

Functional enrichment analysis was performed using the Database for Annotation, Visualization and Integrated Discovery (DAVID) version 6.8 (Huang et al., 2008) to understand the biological meaning and functional grouping of the proteins coded by the genes entering the modules most significantly related to W and PC. Moreover, this analysis may be relevant to identify potential pathways involved in molecular processes associated with the WS/WB occurrence. More in detail, each gene list belonging to the modules selected for functional analyses has been individually analyzed using the Functional Annotation Clustering tool by considering the Biological processes (BP), Molecular function (MF), and Cellular components (CC) GO categories included in the DAVID Knowledgebase for the gene functional characterization. Besides, KEGG pathways (Kanehisa and Goto, 2000) and UP_KEYWORDS categories have been considered for the enrichment analysis. For the present analysis, a Benjamini-adjusted *p*-value of 0.05 was chosen as the significance threshold to identify the most significant functional categories.

The functional characterization of the gene modules most strongly associated with the traits of interest was also performed using “ClueGo” (Bindea et al., 2009), a Cytoscape software 3.8.2 plugin (Shannon et al., 2003). First of all, we individually exported the selected module gene lists to the Cytoscape software by using the export Network To Cytoscape function (“WGCNA” package). Secondly, we used the “GeneMANIA” plugin (Montojo et al., 2010) to create the gene networks in Cytoscape, and then “ClueGo” plugin to obtain a functional characterization of the corresponding modules. Besides, the plugin displayed an “annotation network” by clustering functional terms in different groups based on their similarities. To do this, the plugin calculates the term-term similarity using the corrected kappa statistic, which determines the association strength between the terms (Bindea et al., 2009). Also, this plugin provided an insightful view of functional interrelations by grouping similar terms by the same color (Bindea et al., 2009). In the ClueGO analysis, the *p*-value was adjusted using the Benjamini–Hochberg method, and a threshold for significance of *p* < 0.05 was chosen.

Homo sapiens was used as the reference organism for both DAVID and Cytoscape enrichment analyses.

### Hub Gene Analysis

The hub genes analysis was performed using “cytoHubba” (Chin et al., 2014), a Cytoscape plug-in that allowed us to identify the most interconnected genes (i.e., the hub genes) belonging to each gene network considered in the present study (i.e., the turquoise and yellow2 modules). In particular, cytoHubba enabled us to explore important nodes in the considered biological networks using the Maximal Clique Centrality (MCC) algorithm, developed by Chin et al. (2014), as a topological analysis method. In detail, the MCC algorithm identifies hub nodes by calculating the number of “maximal cliques,” which represents how many connections the node displays. Then, the software created the hub gene network, in which nodes are colored from red to yellow depending on their importance (the most important in dark red vs*.* less important in light-yellow).

## Results

### Co-expression Network Construction and Modules Detection

In the current research, two new traits not yet investigated in our previous work (i.e., the breast width and the total crude protein content of PM muscles) were selected and analyzed using the same methodological approach already reported in Bordini et al. (2021). In particular, we decided to focus our investigation on two traits that can be considered phenotypical markers of high breast yield (i.e., the breast width; W) and muscle degeneration (i.e., the total crude reduced protein content of breast muscle; PC) which usually characterize the WS/WB affected fillets. Then, we analyzed the modules that were characterized by the highest absolute value of correlation with the considered traits of W and PC, namely the yellow2 and turquoise modules. In particular, the yellow2 (named as “width module” hereafter) was found positively associated with W (r = +0.92; *p* < 0.001), while the turquoise module (named as “protein content module” hereafter) was negatively related to PC (*r* = −0.90; *p* < 0.001). Thus, due to their highest absolute value of correlation with the considered traits, the width and protein content modules have been selected for the following analysis. The correlation values between each module and the two considered traits (W and PC) are reported in Supplementary Table S1.

**FIGURE 1:**
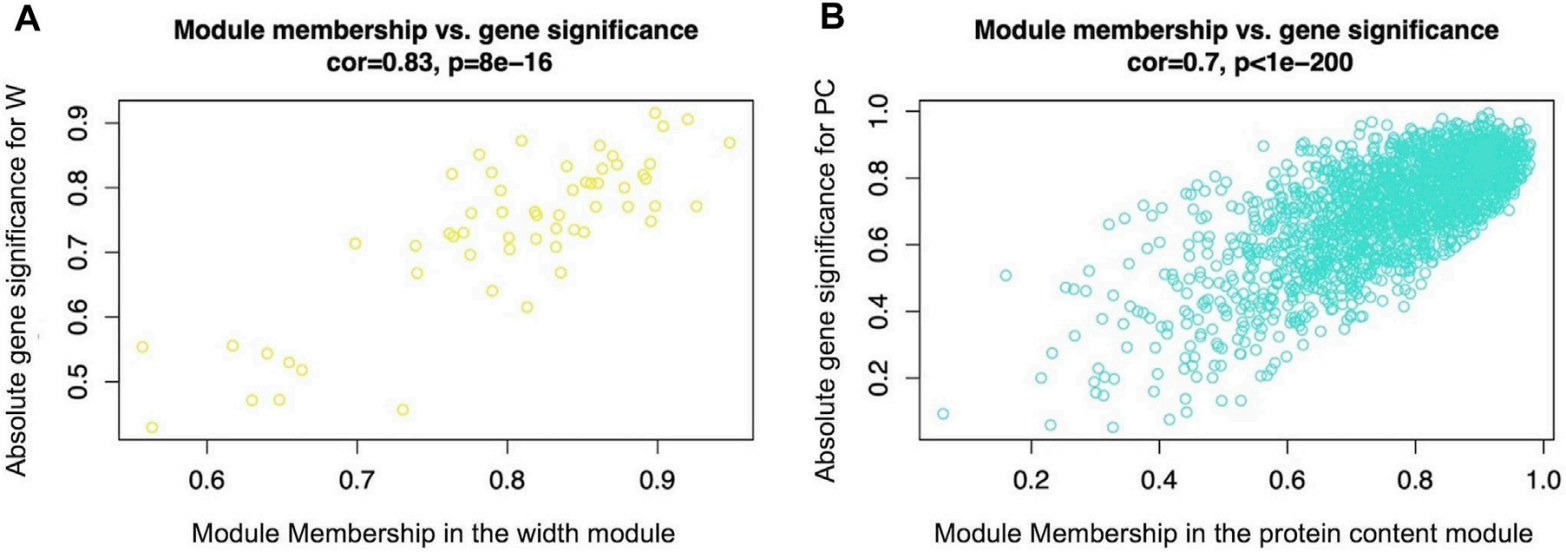
Scatterplot of absolute gene significance (GS, *y*-axis) plotted against the gene module membership (MM, *x*-axis) of the modules selected for further analysis: the width module Figure 1**(A)**, which was the module most significantly related to breast muscle width (W trait) (evaluated in 52-day-old broilers’ *Pectoralis major* muscles), and the protein content module **(B)**, which was the most significant module related to breasts protein contents (PC trait) (evaluated in 52-day-old broilers’ *Pectoralis major* muscles).

Figure 1 reports the WGCNA outputs representing the absolute Gene Significance (GS) of genes belonging respectively to the width (Figure 1A) and protein (Figure 1B) modules, plotted against the corresponding gene module membership (MM). The graphs show that genes characterized by a high absolute value of GS also displayed a high gene MM value. The lists of GS values and the relative *p*-value between all genes and the considered traits (W and PC) are reported in Supplementary Table S2. Also, the complete lists of gene MM values and the relative *p*-values of all genes considered for the present analysis are reported in Supplementary Table S3.

### DAVID Functional Annotation

The lists of genes entering in the width and protein content modules were individually submitted to DAVID online tool to perform functional enrichment analysis. Functional categories of both modules identified as significant analysis are reported in Table 1. With reference to the width module, the “hsa04120: Ubiquitin mediated proteolysis” KEGG pathway was found as unique significant functional term (*p* < 0.01). Considering the protein content module, instead, several significant function terms have been identified, as reported in Table 1. Some of the GO terms correlated with the WS/WB defects were enriched with the “positive regulation of GTPase activity” and “collagen catabolic process,” along with the “ECM-receptor interaction” and “PI3K-Akt signaling pathway” as KEGG pathways. Both for the width and protein content modules, the detailed results obtained by the DAVID Annotation Tool are reported in Supplementary Table S4.

**TABLE 1:** The significantly enriched GO terms, KEGG pathways and UP_KEYWORDS identified by DAVID tools considering the genes comprised in the width (yellow2) and protein content (turquoise) modules and whose mRNA level covaried with the crude protein content and breast width in normal and White Striping/Wooden Breast affected *Pectoralis major* muscles in 52-day-old broilers. Benjamini-adjusted *p* value < 0.05 was chosen as the significance threshold.

Width module (yellow2)			
Category	Term	Gene count	*p* value
KEGG	hsa04120: Ubiquitin mediated proteolysis	10	0.006
**Protein content module (turquoise)**			
**Category**	**Term**	**Gene count**	***p* value**
KEGG	hsa04510: Focal adhesion	54	5.55E-10
	hsa04512: ECM-receptor interaction	29	2.33E-07
	hsa04151: PI3K-Akt signaling pathway	64	6.37E-0.6
GOTERM_BP	GO:0043547∼positive regulation of GTPase activity	82	3.42E-0.4
	GO:0051056∼regulation of small GTPase mediated signal transduction	30	6.41E-04
	GO:0030574∼collagen catabolic process	17	0.01
GOTERM_MF	GO:0005096∼GTPase activator activity	51	2.73E-05
	GO:0098641∼cadherin binding involved in cell-cell adhesion	44	0.01
GOTERM_CC	GO:0005913∼cell-cell adherens junction	49	5.89E-04
	GO:0005581∼collagen trimer	20	0.002
UP_KEYWORDS	Glycoprotein	427	1.29E-08
	GTPase activation	34	1.16E-04
	Hydroxylation	20	8.39E-04
	Collagen	20	0.001

### ClueGO Enrichment Analysis

**FIGURE 2:**
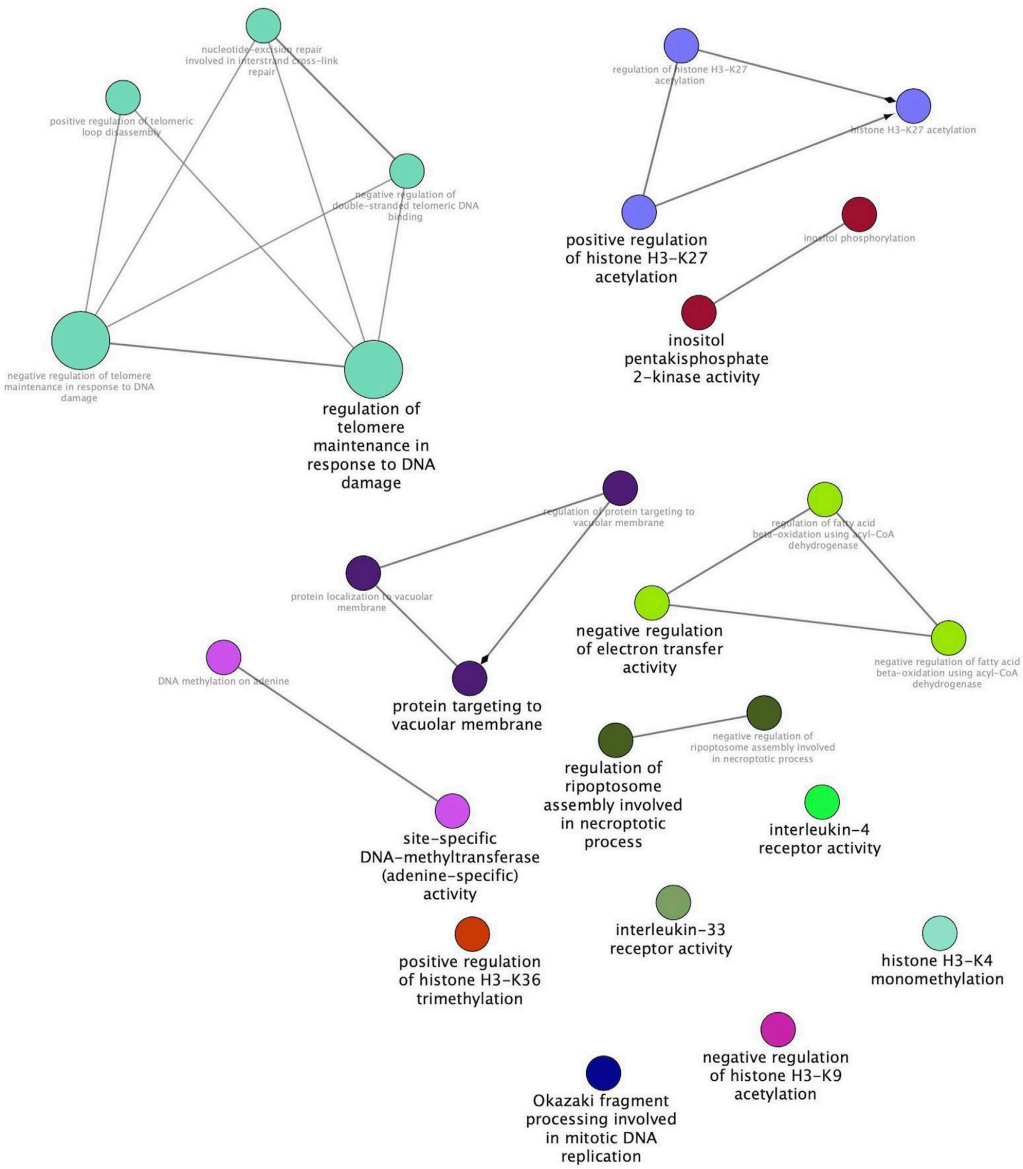
Width module functional network. This image shows functional terms identified by ClueGO, considering genes belonging to the width module and whose mRNA level covaried with the breast width in normal and White Striping/Wooden Breast affected *Pectoralis major* muscles in 52-day-old broilers. Connections show how functional categories interact with each other. Terms belonging to the same functional group are colored with the same color. Black bold terms indicate the leading group term identified by the highest level of significance, using the Benjamini–Hochberg *p*-value and setting *p* < 0.05 as the significant threshold.

**FIGURE 3:**
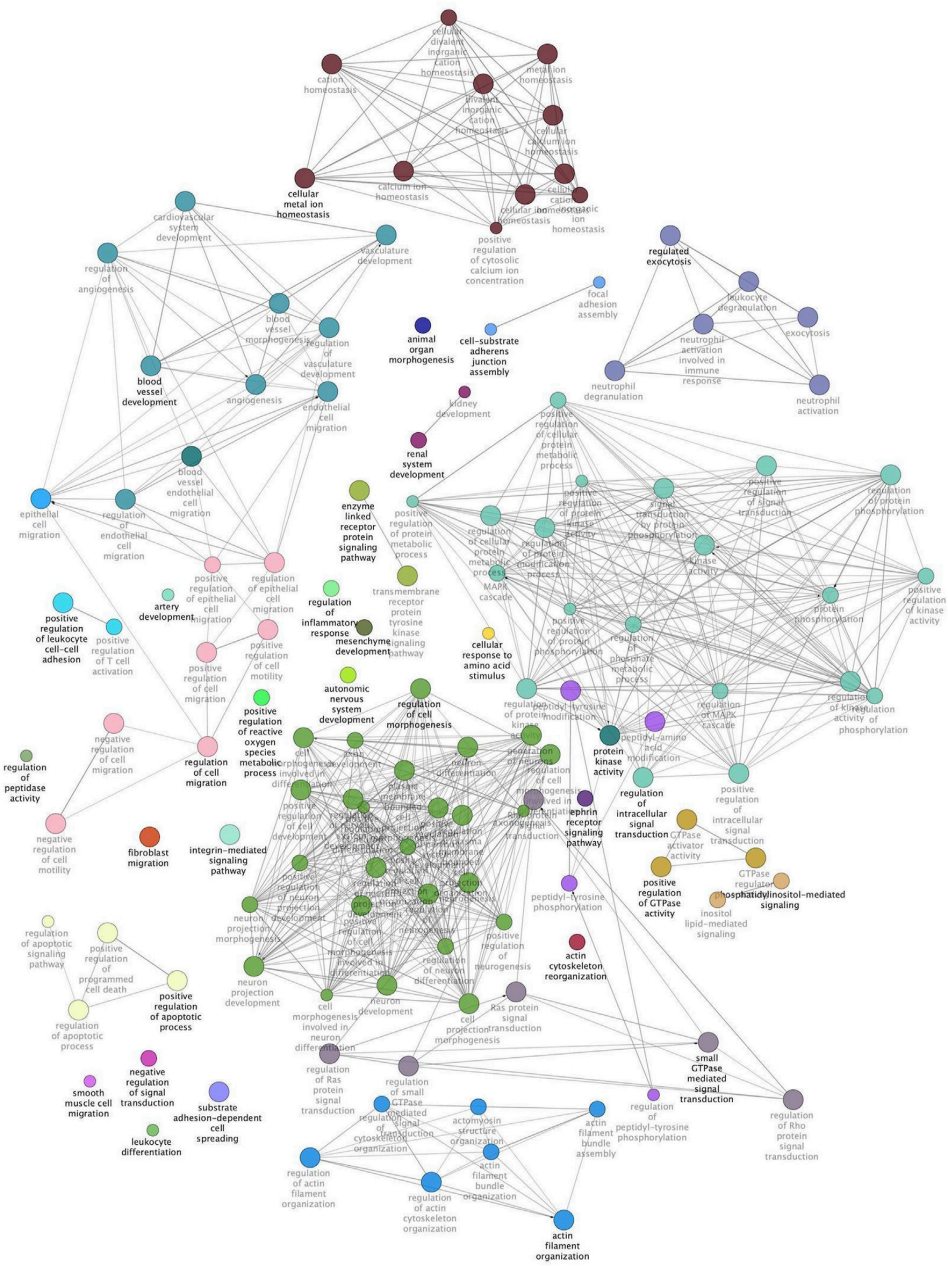
Protein content module functional network. This image shows functional terms identified by ClueGO considering genes belonging to the protein content module and whose mRNA level covaried with the crude protein content in normal and White Striping/Wooden Breast affected *Pectoralis major* muscles in 52-day-old broilers. Connections show how functional categories interact with each other. Terms belonging to the same functional group are colored with the same color, while terms belonging to two different functional groups have both colors of groups they belong to. Black bold terms indicate the leading group term identified by the highest level of significance, using the Benjamini–Hochberg *p*-value and setting *p* < 0.05 as significant threshold.

The same modules were subsequently submitted to Cytoscape with the aim of deepening the enrichment analysis of the width and protein content modules and showing the functional categories interconnections. Figure 2 reports the ClueGO enrichment analysis for the width module. This analysis has shown several genes enriched in categories linked to the interleukin-4 receptor activity, negative regulation of electron transfer activity, and regulation of Ripoptosome assembly involved in the necroptotic process. Figure 3 shows the results obtained by analyzing genes belonging to the protein content module. Among the functional categories identified by ClueGO for this module, several terms were the same found as significant by DAVID tools, such as the GTPase activator activity, focal adhesion assembly, phosphatidylinositol 3-kinase signaling. In addition, the present analysis showed different GO terms related to the regulation of actin cytoskeleton organization, blood vessel development, regulation of fibroblast migration, and regulation of apoptotic signaling pathway.

All the data obtained by the ClueGO analysis for both considered modules are reported in Supplementary Table S5.

### Detection of Hub Genes

Using the Cytoscape plugin “cytoHubba” we performed a hub gene analysis of selected modules that allowed us to identify genes that may play a pivotal role in the biological networks significantly related to the W and PC traits: the width and protein content modules, respectively. Indeed, this analysis allowed us to identify genes characterized by a high degree of interconnection with other genes belonging to the network (i.e., “hub genes”).

**FIGURE 4:**
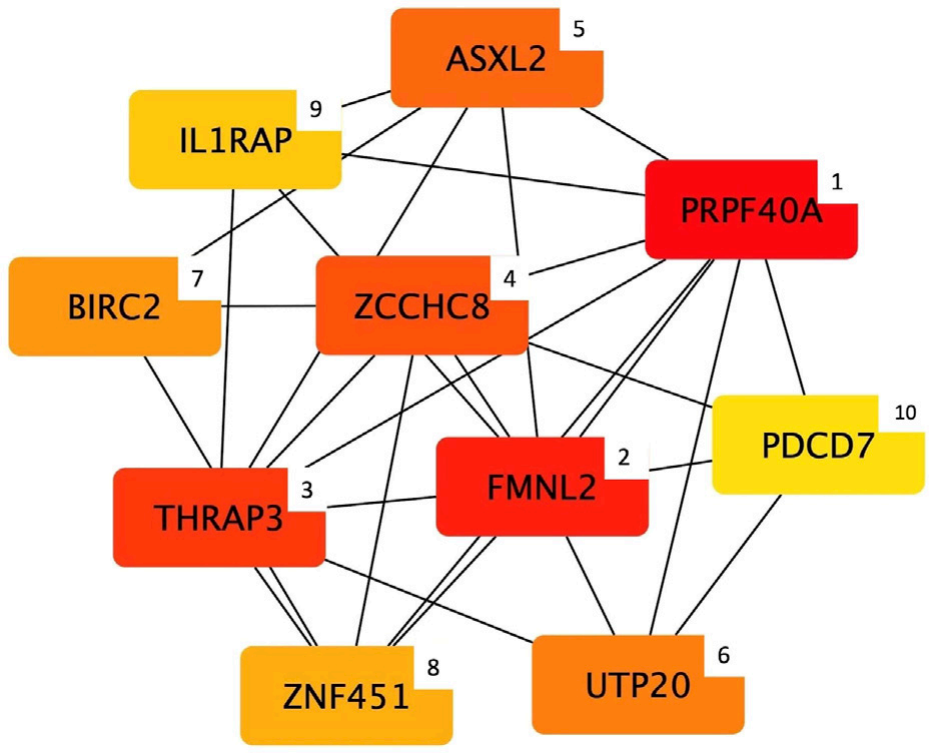
Top 10 hub genes entering the width module (i.e., group of co-expressed genes and whose mRNA level covaried with the breast width in normal and White Striping/Wooden Breast affected *Pectoralis major* muscles in 52-day-old broilers. Connections show how functional categories interact with each other). The cytoHubba output shows hub nodes with the most significant MCC values (i.e., genes characterized by the highest number of connections in the considered network). Besides, the plugin classifies the 10 hub genes by ranking them considering their MCC value and using a color-ranging scale: from the dark red (most important hub gene) to the light-yellow (less important hub gene), also indicated by a numerical scale from 1 (most important) to 10 (less important).

Considering the width module, cytoHubba detected 10 genes (Figure 4) that can be considered hub nodes: *Pre-mRNA processing factor 40 homolog A* (*PRPF40A*), *Formin like 2* (*FMNL2*), *Thyroid hormone receptor associated protein 3* (*THRAP3*), *Zinc finger CCHC-type containing 8* (*ZCCHC8*), *ASXL transcriptional regulator 2* (*ASXL2*), *UTP20 small subunit processome component* (*UTP20*), *Baculoviral IAP repeat containing 2* (*BIRC2*), *Zinc finger protein 451* (*ZNF451*), *interleukin 1 receptor accessory protein* (*IL1RAP*), and *Programmed cell death 7* (*PDCD7*).

**FIGURE 5:**
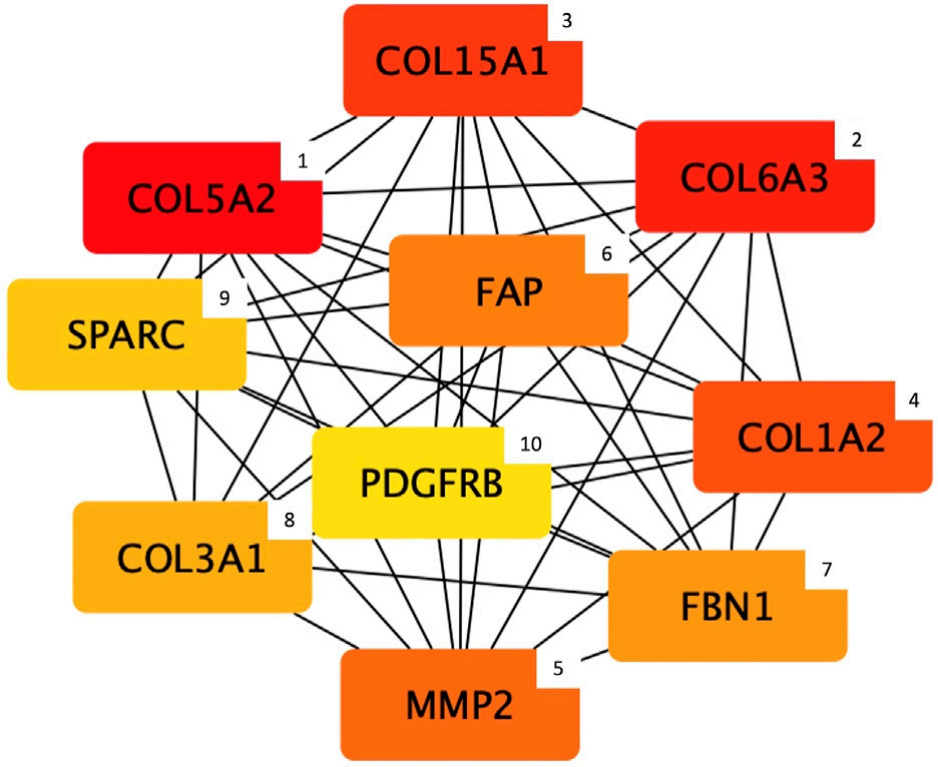
Top 10 hub genes entering the protein content module (i.e., group of co-expressed genes and whose mRNA level covaried with the crude protein content in normal and White Striping/Wooden Breast affected *Pectoralis major* muscles in 52-day-old broilers. Connections show how functional categories interact with each other). The cytoHubba output shows hub nodes with the most significant MCC values (i.e., genes characterized by the highest number of connections in the considered network). Besides, the plugin classifies the 10 hub genes by ranking them considering their MCC value and using a color-ranging scale: from the dark red (most important hub gene) to the light-yellow (less important hub gene), also indicated by a numerical scale from 1 (most important) to 10 (less important).

Regarding the protein content module, the output of the hub gene analysis is reported in Figure 5. Intriguingly, most of the genes identified as hub nodes by cytoHubba encode for proteins related to the connective tissue and extracellular matrix (ECM) composition and organization, which are: *Collagen type V alpha 2 chain* (*COL5A2*), *Collagen type VI alpha 3 chain* (*COL6A3*), *Collagen type XV alpha 1 chain* (*COL15A1*), *Collagen type I alpha 2 chain* (*COL1A2*), *Matrix metallopeptidase 2* (*MMP*), *Fibroblast activation protein alpha* (*FAP*), *Fibrillin 1* (*FBN1*), *Collagen type III alpha 1 chain* (*COL3A1*), *Secreted protein acidic and cysteine rich* (*SPARC*), and *Platelet derived growth factor receptor beta* (*PDGFRB*).

## Discussion

The present research explored new patterns of co-expressed genes not yet considered in our previous study (Bordini et al., 2021) by investigating two PM muscles traits (i.e., breast width- W, and protein content- PC) that were found in Zambonelli et al. (2016) to be significantly associated with the WS/WB onset, and that have not yet been analyzed in depth. The traits examined in this study can be considered markers of the modern broiler growth rate and of the meat quality impairment characterizing the affected fillets. Indeed, the breast width (W) is indicative of the high breast yield of the fast-growing broilers, and the reduced total crude protein content (PC) depicts the impaired composition (as a result of the extensive necrosis of the fibers) characterizing abnormal fillets. Hence, we decided to investigate these traits to better clarify and bring new knowledge on key pathways and molecular players (i.e., hub genes) potentially associated to the complexity of mechanisms underlying these abnormalities. For this purpose, the WGCNA approach allowed us to identify molecular patterns and their most interconnected genes that may have a role in the pathophysiological processes underlying the phenotypic characteristics of PM muscle affected by WS and WB. In fact, the enrichment analysis carried out considering the lists of genes belonging to the width and protein content modules (i.e., the two modules most significantly correlated to the W and PC traits respectively) has provided biological insights for each module examined.

Considering the width module, the “ubiquitin-mediated proteolysis” was the unique functional category identified as significant by DAVID tools. In accordance with this outcome, genes involved in ubiquitin-specific proteases have been found differentially expressed in myopathic affected broilers, thus suggesting their involvement in the higher level of degradation of muscular proteins featuring the PM of affected chickens when compared to the normal counterpart (Kuttappan et al., 2017; Malila et al., 2020; Ayansola et al., 2021; Maharjan et al., 2021). The ubiquitin proteolytic system consists of a cascade of molecular events involved in a broad variety of cellular processes, with particular reference to the degradation of aberrant proteins (Glickman & Ciechanover, 2002; Wertz & Dixit, 2008; Berner et al., 2018). Interestingly, the ubiquitination of aberrant proteins is in line with the hypothesis suggested in our previous paper where it was hypothesized that the endoplasmic reticulum (ER) stress induced by the accumulation of misfolded/unfolded proteins may lead to the cascade of cellular events underlying the onset of these (Bordini et al., 2021). However, it could not be excluded that the activation of this process may result from the myofiber’s alterations characterizing the WS/WB progression. Thus, further studies will be necessary to assess if these conditions could be considered the cause or the effect of these breast abnormalities complex picture.

Moreover, the protein ubiquitination mechanism has recently been reported as a key regulatory process influencing numerous aspects of pathways involved in the repair of damaged DNA (Ghosh and Saha, 2012). Thus, ubiquitination mechanisms could be linked to the functional category “regulation of telomere maintenance in response to DNA damage” identified by ClueGO. Oxidative stress has been demonstrated to accelerate telomere shortening in humans and mice (Barnes et al., 2019). More in detail, oxidative stress in cultured human fibroblasts resulted in increased shortening of telomeres, as well as mitochondrial oxidative stress was shown to induce telomere erosion in human cardiovascular disease (Gordona et al., 2022). Similarly, a recent study by Hubert and Athrey (2022) pointed out the potential relationship between mitochondrial dysfunction, oxidative stress, and the longevity-assurance processes (a complex network of processes linked to the cell maintenance and repair mechanisms including telomere length) (Rattan, 1998) in fast-growing broilers. In light of the above, it could be hypothesized that, similarly to what was evidenced in human and animal models, the accumulation of oxidative damage (for instance due to mitochondrial dysfunction) may determine telomere shortening rates, thus leading to faster cellular senescence. In this scenario, this outcome seems to evidence that telomeres maintenance, as well as the response to DNA damage, could be potentially involved in the complexity of WS/WB underlying mechanisms. As far as we know, this is the first research evidencing a potential association between the telomere maintenance in response to DNA damage with the mechanisms probably involved in the WS/WB occurrence.

With regard to the mitochondrial dysfunction, Papah et al. (2017) evidenced mitochondrial architecture alteration (i.e., disintegration of mitochondrial cristae) in degenerating myofibrils of six-weeks-old wooden breast chickens. The same Authors suggested that alterations of mitochondrial morphology and cristae disruption may lead to an impaired bioenergetics balance of breast muscle fibers, resulting in increased susceptibility to muscle damage. In agreement with this hypothesis, since the mitochondrial electron transport chain is localized in the mitochondrial cristae (Cogliati et al., 2021), our results evidencing the “negative regulation of electron transfer activity” as a significant functional category seem to agree with the findings obtained by Papah et al. (2017). Thus, this result supports the hypothesis of a potential implication of mitochondrial dysfunction in the cascade of events involved in the WS/WB occurrence and/or progression. However, additional studies would be necessary to verify if the alterations in mitochondrial electron transfer activity may be one of the primary causes of abnormal modern broilers’ PM.

Moreover, the ClueGO enrichment analysis of the genes comprised in the width module confirmed the relevance of the muscular inflammation status as one of the main phenomena taking place in WS/WB fillets (Zambonelli et al., 2016; Papah et al., 2017; Xing et al., 2021), and supposedly involved in the activation of signaling pathways leading also to the induction of regenerative processes that characterize myopathic breasts. In fact, ClueGO also identified the “interleukin-4 receptor activity” as a significant functional category. In particular, the muscle-secreted interleukin 4 (IL4) is a cytokine involved not only in the inflammatory response as an anti-inflammatory agent (Gadani et al., 2012) but also in the muscle regeneration process (Guerci et al., 2012; Yang & Hu, 2018; Costa et al., 2021): injured muscles release IL4 and other cytokines (e.g., interleukin 6, IL6; tumor necrosis factor, TNF) as signals in response to muscular damage in hypertrophic muscles with the aim of facilitating the myoblasts migration and their fusion into myotubes (Nierobisz et al., 2008; Świerczek-Lasek et al., 2019). In agreement with these statements, Pampouille et al. (2019) reported that cytokines released during inflammatory response could play a relevant role in muscle regeneration of myopathic chickens by promoting satellite cells (i.e., muscle stem cells; SC) proliferation and differentiation.

Referring to the multiple aspects of the inflammatory condition of the affected PM muscles, ClueGO has also evidenced the “regulation of Ripoptosome assembly involved in the necroptotic process” functional category, which is related to a particular type of necrosis, the necroptotic process. Interestingly, this process is an inflammation-induced form of necrosis initiated by the TNF cytokine secretion (Feoktistova and Leverkus, 2015; Liu et al., 2016) well-described in muscular dystrophies affecting humans, such as the Duchenne Muscular Dystrophy (Morgan et al., 2018; Sreenivasan et al., 2020). Accordingly, the involvement of the TNF-induced necroptosis in the muscle-wasting condition characterizing abnormal chickens’ breasts can be supported also by our results.

Concerning PC trait, the results obtained from the functional enrichment analysis of the protein content module evidenced that genes of this module are functionally related to the reorganization of the actin cytoskeleton. This process is well represented by two of the significant functional categories identified in this study: the “positive regulation of GTPase” and the “actin cytoskeleton organization”. In this regard, several studies highlighted the relevant role of the GTPase superfamily in the actin organization (Matsumoto et al., 1997; Gu et al., 2014) and Mutryn et al. (2015) found several proteins belonging to the GTPase family as up-regulated in woody breasts when compared with fillets exhibiting normal appearance. The same Authors hypothesized that the altered expression of GTPase genes may be linked to the phenomena of disruption and subsequent repair of the actin cytoskeleton manifested in abnormal breasts (Mutryn et al., 2015). Besides having a role in the re-organization of the altered sarcomere architecture characterizing muscles affected by the growth-related myopathies (Velleman et al., 2018), the rearrangement of the actin cytoskeleton has an important role in modulating angiogenesis (Bayless et al., 2011). In this regard, the “blood vessel development” was found among the functional terms identified by ClueGO. Noteworthy, compromised angiogenesis is widely considered as one of the potential causative/major predisposing factors at the basis of the occurrence of growth-related myopathies, as an inadequate development of blood vessels in PM could lead to impaired oxygen and nutrient supply at the muscular level and impaired waste removal (Sihvo et al., 2018; Brothers et al., 2019; Petracci et al., 2019). A large body of literature detected in WS/WB affected muscles consistent changes in the expression level of genes related to the regulation of angiogenesis (Mutryn et al., 2015; Abasht et al., 2016; Zambonelli et al., 2016; Sihvo et al., 2018; Marchesi et al., 2019; Ayansola et al., 2021), suggesting that impaired angiogenesis is a common feature in WS/WB affected PM.

In terms of regulation of the angiogenetic process, genes belonging to the protein content module were also functionally annotated with the “PI3K-Akt signaling pathway.” This pathway plays a critical role in many cellular functions and biological processes including not only angiogenesis but also a wider domain of biological actions concerning proliferation, metabolisms and apoptosis (Bader et al., 2005). In particular, the PI3K pathway influences the endothelial cells’ growth by mediating the vascular endothelial growth factor (VEGF) (Karar and Maity, 2011). These results, considered with the above-mentioned blood vessel development category, strengthen the hypothesis of potential involvement of pathways regulating the angiogenesis process in relation to the reduced vascularization characterizing modern broilers’ PM. Moreover, it is worth mentioning that the PI3K/Akt can also inhibit the expression of genes encoding for the E3 ubiquitin ligases that, as previously stated, regulate the ubiquitin-mediated protein degradation (Yoshida and Delafontaine, 2020). Thus, the functional analysis of the investigated modules allowed us to assume that the traits considered as markers of the WS and WB disorders (i.e., breast width and breast protein content) are associated with groups of co-expressed genes interconnected between each other (for example considering the PI3K/Akt and the ubiquitin-mediated proteolysis). Hence, it could be hypothesized that these groups of genes may contribute together to build the complex pathological framework characterizing WS/WB defects.

Most importantly, the weighted gene network analysis allowed us not only to detect gene expression patterns (i.e., modules) significantly related to the phenotypic variability of the considered traits in normal and WS/WB affected PM muscles, but also to identify central players within the considered modules: the hub genes. Indeed, these genes are characterized by a high number of interactions with other genes belonging to the same network, and generally have crucial roles in regulating biological processes (Yu et al., 2017). Therefore, since hub genes can be considered key regulators in their biological networks (Langfelder et al., 2013), which in turn are significantly related to phenotypic traits considered as indicative markers of WS/WB occurrence, their involvement in the phenotypic variability that characterizes breast muscles affected by WS and WB defects might be hypothesized.

In this context, *BIRC2* is one of the top 10 hub genes identified by CytoHubba for the width module. This gene, also known as *cIAP1* (Cellular Inhibitor of Apoptosis Protein 1), encodes for the homonymous protein that belongs to a group of anti-apoptosis proteins: the IAPs (i.e., inhibitors of apoptotic proteins) (Li et al., 2011). In general, this group of proteins exerts an anti-apoptotic role by inhibiting the caspases signaling system during the apoptosis process (Budihardjo et al., 1999; Fan et al., 2005; Li et al., 2011) and, particularly, it has been demonstrated that Birc2 protein has a relevant role in maintaining endothelial cell survival and vascular homeostasis during vascular tissue development of zebrafish embryos (Santoro et al., 2007). The *BIRC2* gene expression is regulated by the activation of the nuclear factor kappa B (NF-κB) pathway, which is involved in the immune and inflammatory responses by regulating pro-inflammatory cytokine production (Lawrence, 2009). However, it has also been demonstrated an anti-apoptotic function of NF-κB through the regulation of the expression level of genes involved in the anti-apoptosis process, such as the IAPs protein family (Dolcet et al., 2005; Lawrence, 2009). As regards NF-κB activation, different mechanisms have been reported in the literature. Among them, the so-called “canonical NF-κB activation” is directly linked to the ubiquitination of the NF-κB inhibitors (IkBs) via the E3 ubiquitin-protein ligases activity (Santoro et al., 2007; Li et al., 2011; Saleem et al., 2013), which could be probably associated with the “ubiquitin-mediated proteolysis” functional category discussed above in this paper.

Based on these considerations, it is important to note that the ER stress in WB/WS muscle may induce the NF-κB activation in the early phase of the unfolded protein response (UPR) (Kitamura, 2011), thus inducing the expression of several anti-apoptotic genes, including *BIRC2* (Brown et al., 2016). These statements support that ER stress occurring at the endothelial level could be one of the factors potentially involved in the cascade of events that result in the onset and/or progression of the growth-related abnormalities. In these conditions of muscular and/or endothelial dysfunctions likely triggering apoptotic events, it could be hypothesized the role of the hub gene *BIRC2* in contrasting the ER-stress apoptosis by acting against the altered cellular functionality induced by the ER stress.

Moreover, the *BIRC2* gene could be in turn regulated by *IL1RAP,* another hub gene belonging to the width module. *IL1RAP* encodes for the interleukin 1 receptor accessory protein: a coreceptor essential for the transmission of interleukin-1 (IL1) signaling (Ågerstam et al., 2015). IL1 is a pro-inflammatory cytokine implicated in several types of cellular stress responses (Goshen and Yirmiya, 2009; Chen et al., 2012) that can also activate the transcription factor NF-κB. As previously discussed, NF-κB regulates in its turn the expression of several genes involved both in the inflammatory and apoptosis processes (Wang et al., 2002). Therefore, *IL1RAP* may be one of the regulators of the NF-κB, ultimately triggering the expression of *BIRC2*, which contrasts the ER stress-induced apoptosis. Thus, these results confirm the impressive inflammatory status and cellular stress response observed in WS/WB affected muscles by previous studies (Kuttappan et al., 2013; Zambonelli et al., 2016; Papah et al., 2018).

In terms of cellular stress responses, the Cytohubba analysis also detected hub genes involved in the molecular response to hypoxia and oxidative stress conditions: the *PRPF40A* and *THRAP3* genes. In fact, in the literature, it is reported that an over-expression of the *PRPF40A* gene is associated with an upregulation of biochemical pathways linked to hypoxia and oxidative stress conditions (Oleksiewicz et al., 2017), and it is reported that *PRPF40A* expression level could be considered a reliable marker of hypoxia-induced stress in human lung cancer (Huo et al., 2019; Ancel et al., 2021). Similarly, the protein encoded by *THRAP3* has a role in the DNA repair process during oxidative stress events (Jungmichel et al., 2013; Vohhodina et al., 2017). Indeed, Jungmichel et al. (2013) demonstrated that the human *THRAP3* gene exerts a critical role in maintaining DNA genomic stability under oxidative stress conditions by regulating alternative splicing events of genes coding for key proteins of the DNA damage response. Therefore, the present results suggest that this gene could participate in the oxidative stress responses characterizing WS/WB breasts (Abasht et al., 2016; Brothers et al., 2019) and, overall, these findings are in line with the hypothesis that hypoxic conditions could be one of the possible causative processes underlying the occurrence of growth-related muscular disorders affecting modern chickens.

Considering the protein content module, most of the genes identified as “hubs” encode for proteins constituting different types of collagens (i.e., collagen type I, III, V, VI, and XV), and that are involved in their assembly (i.e., secreted protein acidic and cysteine-rich; SPARC) and catabolism (i.e., matrix metallopeptidase 2; MMP2). Collagens are the most abundant proteins belonging to the ECM of connective tissues, and they contribute to the proper development and maintenance of musculoskeletal tissues (Bailey, 1985; Velleman, 2015; Gatseva et al., 2019; Mienaltowski et al., 2021). Because of its importance in the ECM structure, alteration in the deposition and assembly of different types of collagens determine numerous congenital myopathies widely described in humans and other species (Ahmad et al., 2020). For instance, mutations in *COL1A2*, *COL3A1*, and *COL5A2* human genes (identified as hub genes in the present research) are at least in part the genetic basis of the Ehlers-Danlos syndrome: a systemic connective tissue disorder caused by defects in fibrillar collagen deposition (Nuytinck et al., 2000; Mao & Bristow, 2001; Malfait et al., 2005). Besides, of particular interest is *COL6A3* gene, encoding for one of the three alpha chains composing type VI collagen, that in turn is associated with Bethlem and Ullrich congenital human myopathies (Bertini and Pepe, 2002). Recent studies hypothesized that this gene could be considered one of the candidate genes potentially involved in the onset of the WS and WB defects (Pampouille et al., 2018). Accordingly, Papah et al. (2018) and Praud et al. (2020) showed an upregulation of *COL6A3* expression in muscles severely affected by both WS and WB, in agreement with the great amount of fibrosis characterizing myopathic muscles. In this respect, numerous Authors identified genes directly involved in fibrosis development as overexpressed in abnormal breasts (Papah et al., 2018; Brothers et al., 2019; Pampouille et al., 2019; Praud et al., 2020).

With regards to the fibrotic conditions of abnormal breasts, *PDGFRB* was found as a further key node in the protein content module gene network. In general, several Authors suggested that the activation of this pathway may participate in the aberrant deposition of ECM components, which in its turn may lead to the impressive fibrosis which is commonly observed in WB (Papah et al., 2018; Pampouille et al., 2019; Malila et al., 2021). Intriguingly, PDGFR-receptors are implicated in the proliferation, differentiation and migration of myogenic cells (Contreras et al., 2021), and a higher expression level of *PDGFRB* was found in developing and regenerative mice’ skeletal muscles (Jin et al., 1991), as well as in WB fillets (Pampouille et al., 2019; Praud et al., 2020).

In accordance with this statement, recent studies suggested that increasing PDGF-receptors expression supports the activation of fibro-adipogenic progenitors (FAPs) that are involved in the ECM remodeling and, more specifically, in the regulation of regeneration and repair processes of the injured muscles (Contreras et al., 2019, 2021). Interestingly, the *FAP* gene, which encodes for a fibroblast activation protein, was found to be one of the hub nodes belonging to the gene expression network negatively related to the PC trait. Intriguingly, several studies identified genes involved in fibroblasts proliferation and function (e.g., FAPs) as differentially expressed in PM exhibiting myopathic conditions, suggesting their involvement in the abnormal collagen deposition (Mutryn et al., 2015; Papah et al., 2018; Praud et al., 2020). In terms of ECM remodeling, the Matrix Metalloproteinases (MMPs) represent a group of endopeptidases primarily involved in the degradation of almost all ECM components, such as the cross-linked collagen and elastin fibers (Gomes et al., 2012) and therefore involved in the ECM remodeling process. In the present study, the *MMP2* gene was detected as another important hub node belonging to the protein content module gene network. This result can be considered particularly interesting in consideration of the findings obtained in our previous study (Bordini et al., 2021) in which Collagen type IV alpha 1 chain (COL4A1) was identified as the most significant hub gene in the considered gene network. In fact, MMP2 is typically associated with the cleavage of the collagen type IV (COL4) (Zeng et al., 1999), the most abundant component of the basement membranes (BMs) of many tissues (e.g., endothelial cells and muscle fibers). Furthermore, the MMP2 activity is indirectly associated with blood vessel development, thus perfectly overlapping with the results obtained by the functional analysis. Indeed, the degradation of COL4 determines the production of bioactive collagen fragments that can inhibit the angiogenesis process, such as the so-called “Arresten” fragment resulting from the degradation of the COL4 non-collagenous domain (Monaco et al., 2006; Aikio et al., 2012). Therefore, it can be hypothesized that alterations in COL4 degradation that leads to the production of anti-angiogenic fragments could be considered likely involved in the compromised vascularization that characterizes breast muscles of modern chickens (Hoving-Bolink et al., 2000; Sihvo et al., 2017).

Within this framework, the Cytohubba Cytoscape plugin identified *SPARC* as another interesting hub gene that might play a role in the cascade of molecular and physiological events characterizing these myopathic conditions. This gene encodes for the homonymous protein that has a critical role in ensuring the proper secretion and assembly of COL4-networks at the BM level in both vertebrates and invertebrate organisms (Chioran et al., 2017). This COL4-network consists of interactions between COL4 heterotrimers (i.e., protomers) forming a polygonal structure that interacts with integrins and serves as a scaffold for the deposition of other ECM components (e.g., laminins and perlecans) (Chioran et al., 2017; Gatseva et al., 2019). Interestingly, SPARC is a transient component of ECM that works as an extracellular chaperone-like protein necessary for the correct integration of COL4 at the BM level (Martinek et al., 2008; Morrissey et al., 2016). Hence, it is important to note that this result could be considered in line with the hypothesis that alteration in the COL4 network assembly and/or in its intracellular accumulation may results in the occurrence of ER stress condition in muscular cells, thus leading to an alteration of the ECM structure and apoptosis process in abnormal breast muscles. Therefore, these results may suggest that this alteration in the protein assembly and/or secretion may be at least partially due to errors in the SPARC chaperone-like activities. Taken together, these results support the hypothesis that alteration in COL4 secretion and/or assembly of the COL4 network resulting from either an abnormal synthesis of the protein (Bordini et al., 2021) or linked to disorders in collagen-associated chaperone activities (e.g., SPARC), could represent a potential pathological mechanism underlying the onset of growth-related abnormalities.

Overall, the present research has allowed us to obtain a more complete understanding of the complex framework of biological and physiological events at the basis of the growth-related myopathies affecting modern chicken hybrids, along with new insights on genes that may have a role in regulating molecular pathways underlying WS/WB occurrence. In fact, through the functional analysis and hub gene identification, the current research allowed us to identify new candidate genes characterized by a high degree of interconnections with other genes belonging to the same expression pattern. Thus, it could be assumed that these candidate genes could play key roles in the pathogenesis of these abnormalities, such as the genes involved in contrasting apoptotic events (e.g., *BIRC2*) and that may be implicated in collagen degradation and assembly (e.g., *MMP2* and *SPARC*). Furthermore, the consistency between the present results and what we have found in our previous study (Bordini et al., 2021) highlights that the traits most significantly associated with the manifestation of WS and WB defects are related in particular to changes in the expression level of genes linked to ER stress condition and ECM remodeling.

In conclusion, the present findings supported the relevance of ER stress condition in association with inflammatory responses, apoptotic events and degenerative processes taking place within the affected PM, as depicted by both the functional and hub gene analyses. These results are also in line with the hypothesis formulated by Lake et al. (2020) suggesting that abnormal lipid accumulation at the perivascular level may lead to ER and mitochondrial stress, thus resulting in the cascade of events involved in the WS/WB occurrence and development. Moreover, these findings may also support our previous hypothesis that an altered architecture and/or degradation of the type IV collagen may be at least in part involved in the WS/WB onset. Indeed, alteration in the assembly of the COL4 network at the BM level could be the cause of ER stress both at the endothelial and muscular levels, thus leading to apoptosis promoted by the UPR activation and that in turn may be responsible for the activation of pro-inflammatory and anti-apoptotic events (e.g., NF-κB pathway). In this process, the involvement of the *SPARC* gene could be hypothesized as a key player in COL4-network stability. Furthermore, it might be speculated that an abnormal degradation of COL4 protein carried out by MMP2, which can determine the production of fragments having anti-angiogenetic activities, might be involved in the vascular damage of PM muscles exhibiting these myopathic conditions. Although the present study seems to corroborate the hypothesis that COL4 may be one of the players involved in the onset of these myopathies, it could not be excluded that its alterations may be the result of the aberrant conditions characterizing abnormal PM muscles at the collagen level. To summarize, the main findings obtained in the present study, and that could be considered remarkable points for a more complete picture of the WS/WB complexity, highlighted molecular pathways directly related to the blood vessel development and cellular responses to several types of stress conditions: oxidative, mitochondrial, and ER stress. Besides, our results identified hub players potentially involved in the occurrence and/or progression of WS/WB, due to their important role in counteracting ER stress-induced apoptosis (*BIRC2* and *ILRAP1*) and ECM composition (*SPARC* and *MMP2*).

## Data Availability

The datasets presented in this study can be found in online repositories. The names of the repository/repositories and accession number(s) can be found below: https://www.ncbi.nlm.nih.gov/geo/, GSE79276.
