# Supplementary material for: Molecular Pathways and Key Genes Associated With Breast Width and Protein Content in White Striping and Wooden Breast Chicken Pectoral Muscle
Source: Front Physiol. 2022 Jul 8;13:936768. doi: 10.3389/fphys.2022.936768 (PMC9304951; doi:10.3389/fphys.2022.936768)
Supplement: Supplementary file 3 [file DataSheet1.pdf]

Supplementary Material

1 Supplementary Tables captions

Supplementary Table S1. Module-trait association values. The table reports Pearson's correlation coefficients measured between each single module (module eigengene; ME) and considered traits: width (W) and protein content (PC).

Supplementary Table S2. Gene significance (GS) of genes referred to the considered traits. The table reports the complete lists of GS values and the relative P-value between all genes and the considered traits: width (W) and protein content (PC).

Supplementary Table S3. Module Membership (MM) of genes belonging to the yellow2 and turquoise modules. The table reports the complete lists of gene MM values and the relative *P-values* of all genes belonging to the two considered modules: yellow2 and turquoise.

Supplementary Table S4. Functional analysis performed using DAVID tools. The table reports the detailed results of the functional classification of the yellow2 and turquoise modules.

Supplementary Table S5. Functional analysis performed using ClueGO Cytoscape plugin. The table reports the functional categories identified by ClueGO for the yellow2 and turquoise modules.
